# Parasitism Shifts the Effects of Native Soil Microbes on the Growth of the Invasive Plant *Alternanthera philoxeroides*

**DOI:** 10.3390/life13010150

**Published:** 2023-01-04

**Authors:** Chaonan Cai, Yingying Zhao, Yongge Yuan, Junmin Li

**Affiliations:** 1 School of Advanced Study, Taizhou University, Taizhou 318000, China; 2 Zhejiang Provincial Key Laboratory of Plant Evolutionary Ecology and Conservation, Taizhou University, Taizhou 318000, China; 3 College of Life Sciences, Taizhou University, Taizhou 318000, China

**Keywords:** biological invasion, *Cuscuta grovonii*, soil microbes, *Alternanthera philoxeroides*, parasitism

## Abstract

Soil microbes play an important role in plant invasion, and parasitic plants regulate the growth of invasive plants. However, the mechanisms by which parasitic plants regulate the effects of soil microbes on invasive plants have not been investigated. Here, we used the invasive plant *Alternanthera philoxeroides* and the holoparasitic plant *Cuscuta grovonii* to test whether and how *C. grovonii* parasitism shifts the effect of native soil microbes on the growth of *A. philoxeroides*. In a factorial setup, *A. philoxeroides* was grown in pots with the presence versus absence of parasitism and the presence versus absence of native soil microbes. The findings showed that native soil microbes increased the biomass and clonal growth of *A. philoxeroides* only in the absence of a parasite, whereas parasitism decreased the biomass and clonal growth of *A. philoxeroides* only in the presence of soil microbes. In addition, the presence of soil microbes increased the deleterious effects of the parasite on *A. philoxeroides*. These results indicate that parasitism can shift the effects of native soil microbes on the growth of the invasive plant *A. philoxeroides*. Our results enrich the understanding of the mechanisms underlying the success of plant invasion.

## 1. Introduction

As the most significant component of global change, biological invasions, that is, the establishment of non-native species outside of their natural range, have become a major threat to biodiversity and ecosystem functions and cause economic loss at an unprecedented rate as a consequence of human activities [1,2,3,4,5]. Alien invasive species can expand only in the invasion area, effectively expel local species, and occupy the entire ecosystem [1,2]. However, the competition and expansion mechanisms of invasive alien plants remain unclear.

Soil microbes are considered to play an important role in successful plant invasion [6,7,8]. Three hypotheses have been proposed to explain the role of native soil microbes in invasion success. The first is the enhanced mutualism hypothesis, which states that plants encounter better mutualists in the exotic range than in the native range, thus facilitating the availability of major plant nutrients and improving plant stress resistance, thereby promoting plant invasion [7,9]. The second is the degraded mutualism hypothesis, which states that invasive plants benefit less from mutualism than native plants, thus reducing the competitiveness and biotic resistance of mycotrophic native communities [10,11]. The third is the accumulation of pathogens hypothesis, which states that exotic invasive plants can accumulate native soil pathogens that may inhibit native plants [12,13]. Moreover, several studies have shown that the effect of native soil microbes on the success of plant invasion can be regulated by various abiotic and biotic factors such as herbivory, water conditions, and soil nutrients [14,15]. For example, Adomako et al. [14] reported that interactions within different soil microbial communities can result in a synergistic effect on plant growth, which depends on environmental conditions (water and nutrient availability). Exploring the factors that influence the effects of soil microbes on plant invasion can help to find an effective method for biological control.

As holoparasites, *Cuscuta* species absorb water and nutrients from their host plants and inhibit their growth [16,17]. *Cuscuta* species prefer invasive plants over native plants [18] and have potential as biological control agents for the prevention and control of alien plant invasion [19]. Recent studies have shown that the parasitism of *Cuscuta* species can indirectly affect the structure and function of rhizosphere soil microbial communities [20,21,22]. Li et al. [23] used fungicide and bactericide to diminish native fungi and bacteria and found that the parasitism of *C. campestris* could benefit the growth of the co-occurring native plant *Coix lacryma-jobi* only in the presence of a full complement of soil fungi and bacteria. However, little has been focused on the effect of parasitic *Cuscuta* species on the effect of native soil microbes on plant invasion success.

*Alternanthera philoxeroides* (Mart.) Griseb. (commonly called alligator weed), as a typical invasive weed with a wide invasive range, has posed a serious threat to agricultural production and ecological balance worldwide [24,25]. In recent years, most studies on *A. philoxeroides* have focused on the plant morphology, geographic distribution, reproductive characteristic, ecological habit, taxonomy, invasive mechanism, and weed management [26,27]. *A. philoxeroides* belongs to the family Amaranthaceae, and arbuscular mycorrhizal root colonization has been observed in all species of this family [28]. Yang et al. [26] found that changes in microbial communities of *A. philoxeroides* rhizospheric soil induced by the parasitism of *C. australis* could negatively affect host plant growth and positively affect native neighbor plant growth. Therefore, we hypothesized that native soil microbes could benefit from the invasion of *A. philoxeroides* based on the enhanced mutualism hypothesis, and that the parasitism of *Cuscuta* species could shift the effects. In this study, we set up a common pot experiment to assess the main and interactive effects of the parasitic plant *Cuscuta grovonii* and native soil microbes on the invasive plant *Alternanthera philoxeroides*. We aimed to determine whether (1) native soil microbes can promote the growth of invasive plants and whether (2) parasitism can shift the effects of native soil microbes on the growth of invasive plants.

## 2. Materials and Methods

### 2.1. Plant Species

*Alternanthera philoxeroides* is a perennial herb of the genus Alternanthera, which belongs to the family Amaranthaceae. It is native to South America and was introduced into China in the 1930s as a forage crop [24,29]. As a typical invasive plant, it can adapt to a variety of habitats, grow rapidly, and has been widely distributed on continents and islands outside Europe and Africa [29,30].

*Cuscuta grovonii* R. Brown is an annual holoparasitic plant belonging to the genus *Cuscuta* of the family Convolvulaceae. In field surveys, *C. grovonii* often infects herbaceous or shrubby plants such as Fabaceae, Asteraceae (Artemisia), and Verbenaceae (Vitex), including the invasive plant *A. philoxeroides* [26,31].

### 2.2. Containers and Substrate

Each of the 20 experimental containers (57 cm long × 39 cm wide × 15 cm deep) was filled with 16,000 cm^3^ of sterilized substrate consisting of a 2:1 mixture of soil and bark (*v*:*v*). The soil was collected from land on a mountain without plant invasion in Jiaojiang District, Taizhou, China. The soil had a pH of 4.53, 11.71 g kg^−1^ organic matter, 0.280 g kg^−1^ total nitrogen, and 1.206 g kg^−1^ total phosphorus. The bark was added to prevent compaction of the soil. Soil and bark sterilization was performed by autoclaving the soil for 1 h at 121 °C.

### 2.3. Experimental Design

The experiment used a split-split plot design consisting of two soil microbial treatments (present and absent, M+ and M−) as the main plots and two parasite treatments (present and absent, P+ and P−) as the split plots. Each treatment had 5 replicates.

To treat the presence of soil microbes, the substrate in the containers was inoculated with non-autoclaved soil at a ratio of 1:20 (*v*:*v*) [32], whereas in absence of soil microbes, the substrate was inoculated with the same volume of autoclaved soil [33].

On 14 June 2022, stolon fragments of *A. philoxeroides* were collected from a field near Taizhou University, Zhejiang Province, China (28°39′ N, 121°23′ E). The stolon fragments with similar-sized thickness, position, and growth were cut into 5 cm stem segments. Two stem segments of similar size were planted in the center of one container. In total, 20 containers were planted with stem segments of *A. philoxeroides*. After one week, a segment with a similar status was reserved for the subsequent experiment, whereas the other segment was removed. Five weeks after transplantation, a 15 cm long stem piece of *C. grovonii* collected from a field near Taizhou University was wound counterclockwise around the stems of the host plant *A. philoxeroides* to allow the parasite to infect the host plant. Non-parasitized *A. philoxeroides* plants were used as the controls. The experiment was conducted in a glasshouse at Taizhou University with a photoperiod of 16 h at 25 °C (day) and 8 h of darkness at 20 °C (night). The light intensity inside the greenhouse was ∼70% of the natural light outside the greenhouse, and the mean relative humidity was 70%. Water was sprayed evenly onto the soil of each container every day to ensure that the soil was consistently moist. Because *A. philoxeroides* exhibited heavy nutrient deficiency, 500 mL of 1 × Hoagland nutrient solution was added weekly to each container until the experimental materials were harvested.

### 2.4. Harvest and Measurements

The *C. grovonii* were harvested 85 days after transplantation by detaching them from *A. philoxeroides*. The number of leaves, ramets, and nodes of *A. philoxeroides* were counted using the counting method. The internode length, stolon length, and stem diameter of *A. philoxeroides* were measured using a ruler and a Vernier caliper. The *A. philoxeroides* plants were then separated into leaves, roots, and stolons, and placed into different envelopes. These plant parts were dried in an oven to a constant weight at 65 °C and then weighed to obtain dry biomass.

### 2.5. Data Analysis

We used soil microbial treatments (present and absent, M+ and M−) as the main plots treatment, parasite treatments (present and absent, P+ and P−) as the split plots treatment and their interactive effect to analyze the split plot design experiment. The response variables included total biomass, leaf biomass, stolon biomass, belowground biomass, aboveground biomass, ramet number, stolon length, internode length, leaf number, node number, stem diameter, and root: shoot ratio. When necessary, data were transformed to meet the assumptions of normality and equal variance. Fisher’s least significant difference (LSD) test was applied to examine the differences among treatments at the 5% significance level. A t-test was used to determine the effects of soil microbes on the biomass of *C. grovonii* and the deleterious effects (DEs) of the parasites on the hosts. The DE was calculated as the difference in total biomass between the parasitized plants and the mean total biomass of the control plants, standardized to the mean biomass of the control plants [18]. According to its value, DE > 0 indicates that parasitism facilitates the growth of the host, DE < 0 indicates that parasitism suppresses the growth of the host, and DE = 0 indicates that parasitism had no effect on the growth rate of the host [18]. All analyses were performed using the Statistical Product and Service Solution (SPSS) software (version 16.0; SPSS Inc., Chicago, IL, USA). In the SPSS statistical analysis, and because the analysis method of the fully random factorial design with repeated measurements is equivalent to the split plot design, we applied the analysis method of fully factorial design to conduct the subsequent analysis in this study [34].

## 3. Results

### 3.1. Effects of Parasites and Soil Microbes on the Biomass of A. philoxeroides

**Figure 1:**
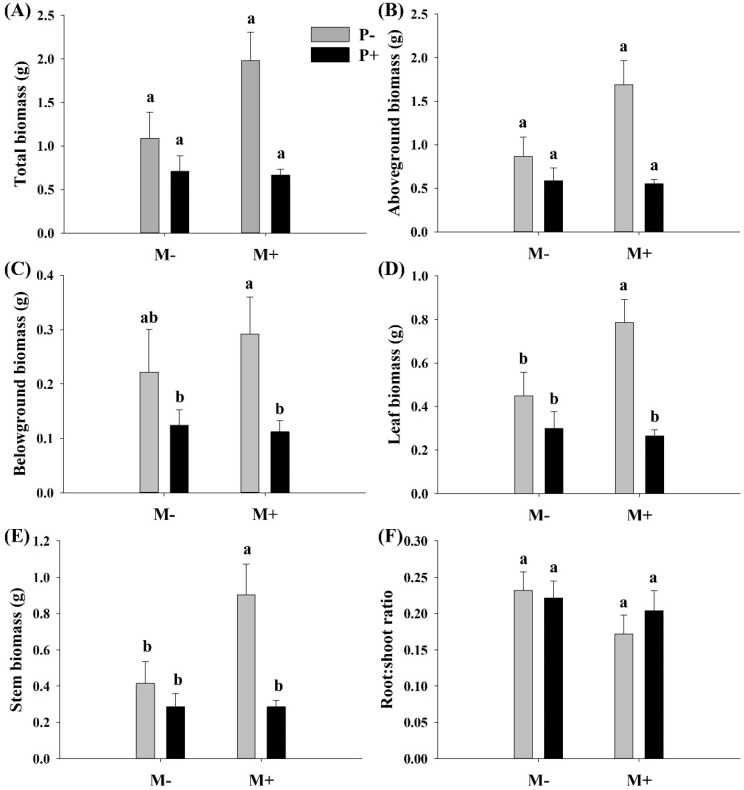
Effect of soil microbes and *Cuscuta grovonii* parasitism on (**A**) total biomass, (**B**) aboveground biomass, (**C**) belowground biomass, (**D**) leaf biomass, (**E**) stem biomass, and (**F**) root: shoot biomass of *A. philoxeroides*. Bars with the same letter do not differ significantly according to the least significant difference (LSD) post hoc test at the 5% level. Values are expressed as the mean ± standard error (n = 5). M+: soil microbe present, M−: soil microbe absent, P+: host *A. philoxeroides* parasitized by *C. grovonii*, and P+: host *A. philoxeroides* not parasitized by *C. grovonii*.

Parasites significantly affected the belowground biomass, leaf biomass, and stem biomass of *A. philoxeroides* but had no significant effect on the total biomass, aboveground biomass, and R/S ratio (Table 1). Soil microbes significantly increased the stem biomass of *A. philoxeroides* but had no significant effect on the total biomass, aboveground biomass, belowground biomass, and R/S ratio (Table 1). There was no significant interactive effect between soil microbes and parasitism on the growth of *A. philoxeroides*, except for leaf biomass of *A. philoxeroides* (Table 1). When soil microbes were present, the parasite significantly decreased the belowground biomass and the leaf and stem biomass of *A. philoxeroides*. However, when soil microbes were absent, the effects of parasites on belowground biomass, leaf, and stem biomass of *A. philoxeroides* were not significant (Figure 1A–F). The effect of soil microbes on the stem and leaf biomass of *A. philoxeroides* was significant only in the absence of parasites (Figure 1D,E). 

### 3.2. Effects of Parasites and Soil Microbes on the Clonal Propagation of A. philoxeroides

**Figure 2:**
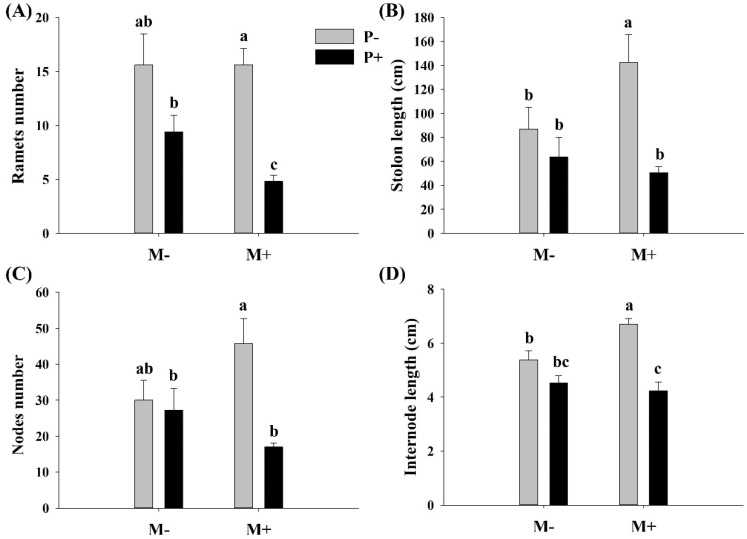
Effect of soil microbes and *Cuscuta grovonii* parasitism on (**A**) the ramet number, (**B**) stolon length, (**C**) node number, and (**D**) internode length of *A. philoxeroides*. Bars with the same letter do not differ significantly according to the least significant difference (LSD) post hoc test at the 5% level. Values are expressed as the mean ± standard error (n = 5). M+: soil microbes present, M−: soil microbes absent, P+: host *A. philoxeroides* parasitized by *C. grovonii*, and P+: host *A. philoxeroides* not parasitized by *C. grovonii*.

Parasitism significantly affected the ramet number, stolon length, internode length, and node number of *A. philoxeroides* (Table 1). The soil microbes did not significantly affect the internode length, node diameter, and stem diameter of *A. philoxeroides*. However, there was a significant interaction effect between parasitism and soil microbes on ramet number, internode length, and node number (Table 1). Specifically, parasites significantly decreased the ramet number, stolon length, node number, and internode length of *A. philoxeroides* only when soil microbes were present (Figure 2A–D). Soil microbes significantly decreased the ramet number of *A. philoxeroides* only in the presence of parasites (Figure 2A), whereas they significantly increased the stolon and internode lengths of *A. philoxeroides* only in the absence of parasites (Figure 2B,D).

### 3.3. Effects of Parasites and Soil Microbes on the Morphology of A. philoxeroides

**Figure 3:**
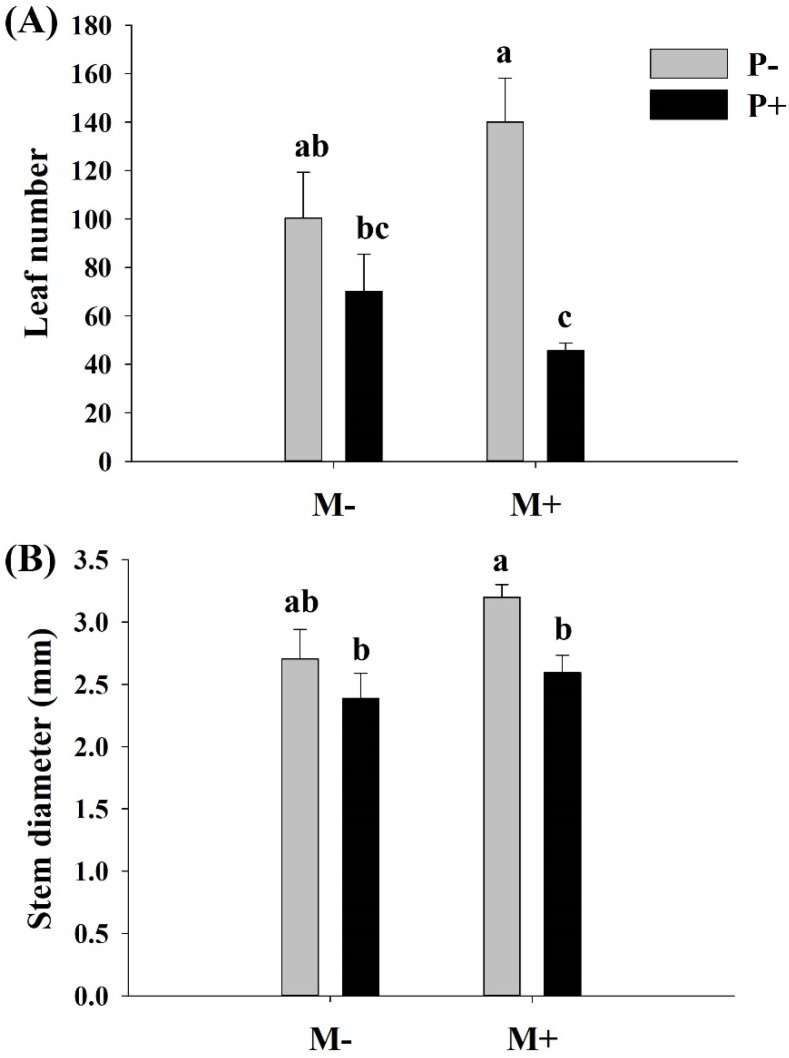
Effects of soil microbes and *Cuscuta grovonii* parasitism on the (**A**) leaf number and (**B**) stem diameter of *A. philoxeroides*. Bars with the same letter do not differ significantly according to the least significant difference (LSD) post hoc test at the 5% level. Values are expressed as the mean ± standard error (n = 5). M+: soil microbes present, M−: soil microbes absent, P+: host *A. philoxeroides* parasitized by *C. grovonii*, and P+: host *A. philoxeroides* not parasitized by *C. grovonii*.

Parasitism, but not soil microbes, significantly decreased the number of leaves and the stem diameter of *A. philoxeroides* (Table 1). There were no significant interactive effects between the soil microbes and parasites on the leaf number and stem diameter of *A. philoxeroides* (Table 1). Parasitism significantly decreased the number of leaves and the stem diameter of *A. philoxeroides* only in the presence of soil microbes (Table 1, Figure 3).

### 3.4. Effects of Soil Microbes on the Biomass of Cuscuta grovonii

**Figure 4:**
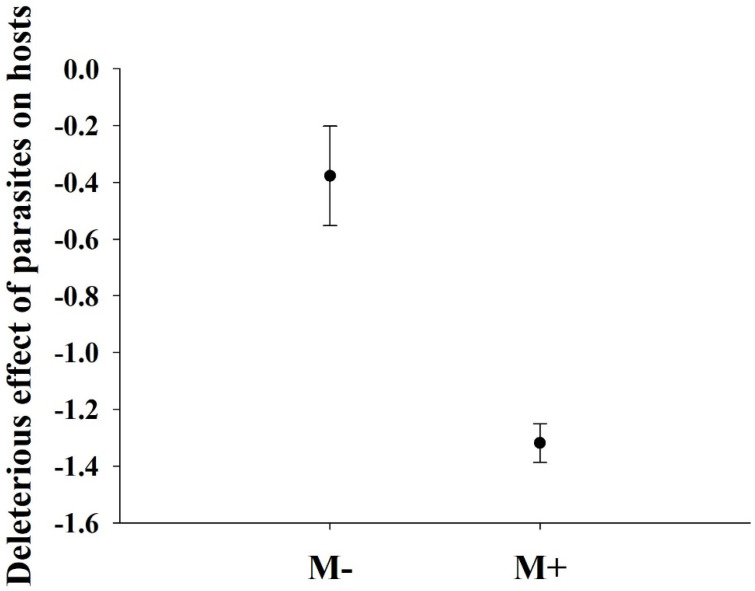
Mean and standard errors of the deleterious effects of parasites on *A. philoxeroides*. Values are expressed as the mean ± standard error (n = 5). M+: soil microbe present and M−: soil microbe absent.

Soil microbes did not significantly affect the biomass of the parasite plant *C. grovonii* (P = 0.078). The deleterious effect of parasites on the growth of *A. philoxeroides* was significantly greater when soil microbes were present than when they were absent (Figure 4).

## 4. Discussion

A comparison of plant performance between unsterilized and sterilized soils was used to evaluate the overall effects of the full soil biota [35]. In this study, we found that native soil microbes promoted the growth of *A. philoxeroides*, including biomass and clonal propagation, indicating that *A. philoxeroides* benefited strongly from native soil microbes in the invaded range. Previous studies have shown that soil microbes promote the invasion of alien plants [8,36]. In addition, research has shown that there is favorable feedback from the soil microbes in the invasion area for the growth of invasive plants, which may be because the natural enemies of the invasive plants did not invade the new habitat with them, and the invasive plants escaped from the soil pathogenic microorganisms in the native soil [8,37,38], or because the invaders formed new mutualism with local soil microbes [8,39]. Colonization of *A. philoxeroides* roots by arbuscular mycorrhizal fungi (AMF) was observed in this study (Figure S1) and supported the enhanced mutualism hypothesis [7,9]. However, further studies are needed to determine the mechanisms underlying the promotion effect of native soil microbes. 

Previous studies have indicated that parasitism negatively affects the growth of invasive species by extracting resources, such as nutrients, water, and organic compounds from the host’s vascular system [18,19,23]. Gao et al. [40] also found that parasitism can affect clonal propagation of clonal plants. In this study, we found that parasitism suppressed the growth and clonal propagation of *A. philoxeroides* only in the presence of native microbes, indicating that soil microbes play a key role in the inhibitory effects of parasitic plants. A similar result has been verified by Li et al. [23] who found the parasitism of *C. campestric* shifts the competition between invasive *Mikania micrantha* and native *Coix. lacryma-jobi* depending on the presence of soil fungi and bacteria. 

Our results showed that parasitism can suppress the effects of native soil microbes on the growth of invasive plants. Such a suppression effect may result from the reduced benefit from soil microbes due to changes in microbial communities, or the fact that part of the benefit was transferred to the parasitic plants. Although direct contact between parasitic plants and the soil ecosystem through roots may be minimal or nonexistent, parasitic plants can have considerable effects on soil organisms [22]. On one hand, parasitism can affect the community of rhizosphere soil microbes by changing the root exudate or by changing the mutualism between the host and soil microbes [22,41]; on the other hand, parasitic plants may affect the community of soil microbes through the inputs of their particularly nutrient-rich litter [42,43]. The changes in soil microbes due to parasitism may reduce the benefits obtained by host plants. Second, *Cuscuta grovonii* obtains all its nutrients from host plants. The benefits provided by soil microbes may be reallocated to *C. grovonii*, thereby indicating a non-significant effect of soil microbes on host plant growth. 

In this study, we also found that soil microbes significantly increased the deleterious effects of parasites on the growth of *A. philoxeroides*. Previous studies have shown that nutrient availability can affect host plant tolerance to biotic stresses [44]. Because soil microbes can enhance nutrient provision to host plants, the deleterious effects of parasites on the growth of host plants may be altered by the presence of soil microbes. In our study, we speculated that the enhanced deleterious effect of parasites on *A. philoxeroides* might have resulted from the fact that native soil microbes promote the growth of host plants, and *C. grovonii* can acquire more resources from the host plant [18,45], thereby causing a more deleterious effect on *A. philoxeroides*. Therefore, we encourage exploration of the potential of parasitic plants to suppress invasive species and develop methods for practical applications in ecological restoration and nature conservation.

## 5. Conclusions

Consistent with our hypothesis, we found that native soil microbes could promote the growth of invasive plants, and that parasitism could suppress the effects of native soil microbes on the growth of invasive plants. In addition, we found that soil microbes enhanced the deleterious effects of parasitism on invasive host plants. Our results suggest that parasitic plants can not only directly affect the growth of invasive plants but can also indirectly interact with soil microbes to affect the growth of invasive plants. This study provides an example of the effects of top-down biotic factors (aboveground parasitic plant and belowground soil microbes) on the growth of invasive plants. Nevertheless, we are still at the beginning of our empirical and applied research, and more invasive and parasitic plant pairs are required to verify this conclusion further.

## Figures and Tables

**Table 1:** Effects of native soil microbe inoculation (M) and infection with the *Cuscuta grovonii* parasite (P) on the growth of *A. philoxeroides*. Bold *p* values indicate *p* < 0.05.

Variables	Source of Variation	d.f.	F Value	*p* Value
Total biomass	P	1	0.045	0.512
	M	1	0.000	0.998
	P × M	1	0.024	0.879
Aboveground biomass	P	1	4.315	0.054
	M	1	0.731	0.405
	P × M	1	0.468	0.504
Belowground biomass	P	1	6.507	0.021
	M	1	0.257	0.619
	P × M	1	0.577	0.459
Leaf biomass	P	1	12.707	0.003
	M	1	3.116	0.097
	P × M	1	4.478	0.050
Stem biomass	P	1	10.875	0.005
	M	1	4.873	0.042
	P × M	1	4.159	0.058
Root: shoot biomass	P	1	0.167	0.688
	M	1	2.795	0.114
	P × M	1	0.702	0.414
Ramets number	P	1	26.817	0.000
	M	1	3.292	0.088
	P × M	1	4.898	0.042
Stolon length	P	1	11.498	0.004
	M	1	1.560	0.230
	P × M	1	4.057	0.061
Internode length	P	1	31.464	0.000
	M	1	3.009	0.102
	P × M	1	7.363	0.015
Nodes number	P	1	8.529	0.010
	M	1	0.268	0.612
	P × M	1	5.774	0.029
Leaf number	P	1	16.586	0.001
	M	1	0.240	0.631
	P × M	1	4.403	0.052
Stem diameter	P	1	6.694	0.020
	M	1	3.915	0.065
	P × M	1	0.650	0.432

## Data Availability

The datasets generated during and/or analyzed during the current study are available from the corresponding author upon reasonable request.

## Supplementary Materials

The following supporting information can be downloaded at: https://www.mdpi.com/article/10.3390/life13010150/s1, Figure S1: Hyphal colonization of *Alternanthera philoxeroides* by AMF magnified 20 times using a stereo microscope (Motic microscope SMZ-168).

## References

[1] Mack R.N., Simberloff D., Lonsdale W.M., Evans H., Clout M., Bazzaz F.A. (2000). Biotic invasions: Causes, epidemiology, global consequences, and control. Ecol. Appl..

[2] Vilà M., Espinar J.L., Hejda M., Hulme P.E., Jarošík V., Maron J.L., Pergl J., Schaffner U., Sun Y., Pyšek P. (2011). Ecological impacts of invasive alien plants: A meta-analysis of their effects on species.; communities and ecosystems. Ecol. Lett..

[3] Liu Y.J., Oduor A.M.O., Zhang Z., Manea A., Tooth I.M., Leishman M.R., Xu X.L., van Kleunen M. (2017). Do invasive alien plants benefit more from global environmental change than native plants?. Glob. Chang. Biol..

[4] Zhang P., Li B., Wu J.H., Hu S.J. (2019). Invasive plants differentially affect soil biota through litter and rhizosphere pathways: A meta-analysis. Ecol. Lett..

[5] Zhou Y., Staver A.C. (2019). Enhanced activity of soil nutrient releasing enzymes after plant invasion: A meta-analysis. Ecology.

[6] Pei Y., Siemann E., Tian B., Ding J. (2020). Root flavonoids are related to enhanced AMF colonization of an invasive tree. AoB Plants.

[7] Yu H., He Y., Zhang W., Chen L., Zhang J., Zhang X., Dawson W., Ding J. (2022). Greater chemical signaling in root exudates enhances soil mutualistic associations in invasive plants compared to natives. New Phytol..

[8] Reinhart K.O., Callaway R.M. (2004). Soil biota facilitate exotic Acer invasions in Europe and North America. Ecol. Appl..

[9] Reinhart K.O., Callaway R.M. (2006). Soil biota and invasive plants. New Phytol..

[10] Stinson K.A., Campbell S.A., Powell J.R., Wolfe B.E., Callaway R.M., Thelen G.C., Hallett S.G., Prati D., Klironomos J.N. (2006). Invasive plant suppresses the growth of native tree seedlings by disrupting belowground mutualisms. PLoS Biol..

[11] Vogelsang K.M., Bever J.D. (2009). Mycorrhizal densities decline in association with nonnative plants and contribute to plant invasion. Ecology.

[12] Eppinga M.B., Rietkerk M., Dekker S.C., de Ruiter P.C., van der Putten W.H. (2006). Accumulation of local pathogens: A new hypothesis to explain exotic plant invasions. Oikos.

[13] Mangla S., Callaway R.M. (2008). Exotic invasive plant accumulates native soil pathogens which inhibit native plants. J. Ecol..

[14] Adomako M.O., Xue W., Tang M., Du D.L., Yu F.H. (2020). Synergistic effects of soil microbes on *Solidago canadensis* depend on water and nutrient availability. Microb. Ecol..

[15] Sveen T.R., Netherway T., Juhanson J., Oja J., Borgström P., Viketoft M., Strengbom J., Bommarco R., Clemmensen K., Hallin S. (2021). Plant-microbe interactions in response to grassland herbivory and nitrogen eutrophication. Soil Biol. Biochem..

[16] Penning S.C., Callaway R.M. (2002). Parasitic plants: Parallels and contrasts with herbivores. Oecologia.

[17] Press M.C., Phoenix G.K. (2005). Impacts of parasitic plants on natural communities. New Phytol..

[18] Li J.M., Jin Z.X., Song W.J. (2012). Do native parasitic plants cause more damage to exotic invasive hosts than native non-invasive hosts? An implication for biocontrol. PLoS ONE.

[19] Yu H., Yu F.H., Miao S.L., Dong M. (2008). Holoparasitic *Cuscuta campestris* suppresses invasive *Mikania micrantha* and contributes to native community recovery. Biol. Conserv..

[20] Bardgett R.D., Smith R.S., Shiel R.S., Peacock S., Simkin J.M., Quirk H., Hobbs P.J. (2006). Parasitic plants indirectly regulate below-ground properties in grassland ecosystems. Nature.

[21] Li J.M., Jin Z.X., Hagedorn F., Li M.H. (2014). Short-term parasite-infection alters already the biomass, activity and function diversity of soil microbial communities. Sci. Rep..

[22] Brunel C., Yang B.F., Pouteau R., Li J.M., van Kleunen M. (2020). Responses of rhizospheric microbial communities of native and alien plant species to *Cuscuta* parasitism. Microb. Ecol..

[23] Li J.M., Oduor A.M.O., Yu F.H., Dong M. (2019). A native parasitic plant and soil microorganisms facilitate a native plant co-occurrence with an invasive plant. Ecol. Evol..

[24] Ye W.H., Li J., Cao H.L., Ge X.J. (2003). Genetic uniformity of *Alternanthera philoxeroides* in South China. Weed Res..

[25] Yin J.L., Hou L., Jiang X.C., Yang J., He Y., Zhou X.K., Zhu X.M., Gong A.D., Zhu Y.X., Chen Z.Y. (2021). Identification and validation of reference genes for quantitative real-time PCR studies in alligatorweed (*Alternanthera philoxeroides*). Weed Sci..

[26] Yang B.F., Zhang X., Zagorchev L., Li J.M., Frey B., Li M.H. (2019). Parasitism changes rhizospheric soil microbial communities of invasive *Alternanthera philoxeroides*, benefitting the growth of neighboring plants. Appl. Soil Ecol..

[27] Dong B.C., Alpert P., Guo W., Yu F.H. (2012). Effects of fragmentation on the survival and growth of the invasive, clonal plant *Alternanthera philoxeroides*. Biol. Invasions.

[28] Arriola L., Niemira B.A., Safir G.R. (1997). Border cells and arbuscular mycorrhizae in four Amaranthaceae species. Phytopathology.

[29] Sainty G., McCorkelle G., Julien M. (1997). Control and spread of alligator weed *Alternanthera philoxeroides* (Mart.) Griseb., in Australia: Lessons for other regions. Wetl. Ecol. Manag..

[30] Holm L.G., Weldon L.W., Blackburn R.D. (1969). Aquatic weeds. Science.

[31] Fang R.C., Lytton J.M., Uzi P., Wu Z.Y., Raven P.H., Hong D.Y. (1995). Cuscuta Linnaeus. Flora of China.

[32] Lau J.A., Lennon J.T. (2011). Evolutionary ecology of plant-microbe interactions: Soil microbial structure alters selection on plant traits. New Phytol..

[33] Borda V., Longo S., Marro N., Urcelay C. (2021). The global invader Ligustrum lucidum accumulates beneficial arbuscular mycorrhizal fungi in a novel range. Plant Ecol..

[34] Zhang W.T., Dong W. (2004). SPSS Statistical Analysis Advanced Course.

[35] Parepa M., Schaffner U., Bossdorf O. (2013). Help from under ground: Soil biota facilitate knotweed invasion. Ecosphere.

[36] Callaway R.M., Cipollini D., Barto K., Thelen G.C., Hallett S.G., Prati D., Stinson K., Klironomos J. (2008). Novel weapons: Invasive plant suppresses fungal mutualists in America but not in its native Europe. Ecology.

[37] Bais H.P., Vepachedu R., Gilroy S., Callaway R.M., Vivanco J.M. (2003). Allelopathy and exotic plant invasion: From molecules and genes to species interactions. Science.

[38] Beckstead J., Parker I.M. (2003). Invasiveness of Ammophila arenaria: Release from soil-borne pathogens?. Ecology.

[39] Whipps J.M. (2001). Microbial interactions and biocontrol in the rhizosphere. J. Exp. Bot..

[40] Gao F.L., Alpert P., Yu F.H. (2021). Parasitism induces negative effects of physiological integration in a clonal plant. New Phytol..

[41] Mishev K., Dobrev P.I., Lacek J., Filepová R., Yuperlieva-Mateeva B., Kostadinova A., Hristeva T. (2021). Hormonomic changes driving the negative impact of broomrape on plant host interactions with arbuscular mycorrhizal fungi. Int. J. Mol. Sci..

[42] Beare M.H., Neely C.L., Coleman D.C., Hargrove W.L. (1990). A substrate induced respiration (SIR) method for measurement of fungal and bacterial biomass on plant residues. Soil Biol. Biochem..

[43] Quested H.M., Cornelissen J.H.C., Press M.C., Callaghan T.V., Aerts R., Trosien F., Riemann P., Gwynn-Jones D., Kondratchuk A., Jonasson S.E. (2003). Decomposition of sub-arctic plants with differing nitrogen economies: A functional role for hemiparasites. Ecology.

[44] Wise M.J., Abrahamson W.G. (2007). Effects of resource availability on tolerance of herbivory: A review and assessment of three opposing models. Am. Nat..

[45] Matzek V. (2011). Superior performance and nutrient-use efficiency of invasive plants over non-invasive congeners in a resource limited environment. Biol. Invasions.

