# Supplementary material for: Parasitism Shifts the Effects of Native Soil Microbes on the Growth of the Invasive Plant Alternanthera philoxeroides
Source: Life (Basel). 2023 Jan 4;13(1):150. doi: 10.3390/life13010150 (PMC9863507; doi:10.3390/life13010150)
Supplement: Supplementary file 1 [file life-13-00150-s001.zip › life-2093308-supplementary.pdf]

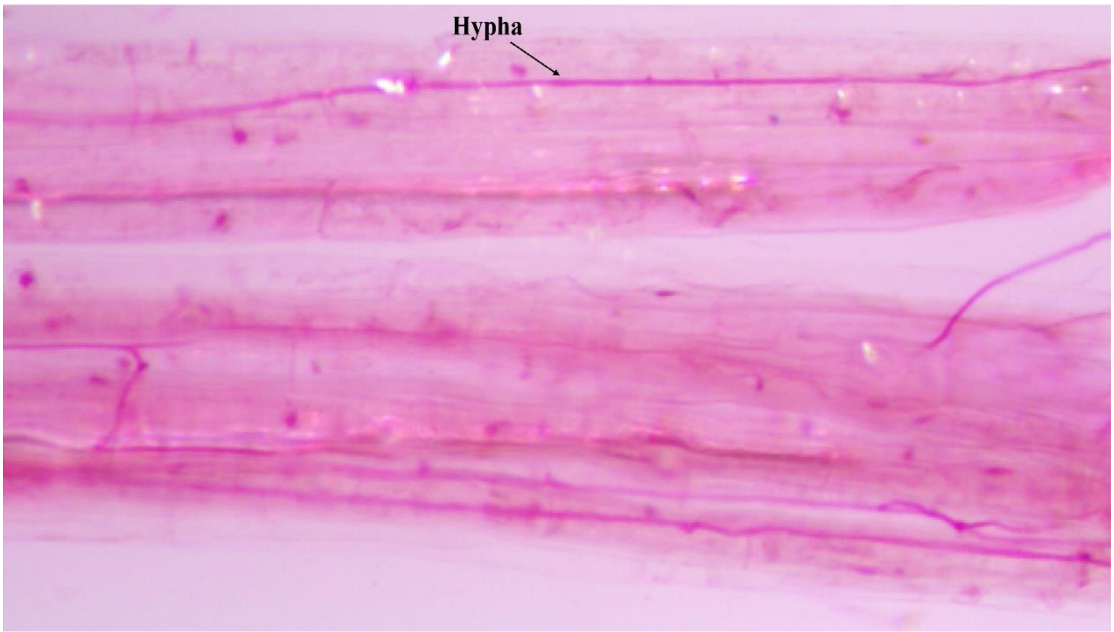


**Figure S1.** Hyphal colonization of *Alternanthera philoxeroides* by AMF magnified by 20 times using a stereo microscope (Motic microscope SMZ-168).
